# Functional characterization of the N-terminal and C-terminal domains of a sesame group II phytocystatin

**DOI:** 10.1186/1999-3110-55-18

**Published:** 2014-02-03

**Authors:** Mei-Ling Cheng, Jason T C Tzen, Douglas J H Shyu, Wing-Ming Chou

**Affiliations:** 1 Graduate Institute of Biotechnology, National Chung-Hsing University, Taichung, 412 Taiwan; 2 Department of Biological Science and Technology, National Pingtung University of Science and Technology, Pingtung, 912 Taiwan; 3 Department of Biotechnology, National Formosa University, Yunlin, 632 Taiwan

**Keywords:** Phytocystatin, Cysteine protease, Papain, Antifungal, *Sesamun indicum*

## Abstract

**Background:**

Phytocystatins are natural inhibitors of cysteine protease, and may regulate endo- or exo-genous proteolytic activities in plants. They are classified into Group I and II differing by the presence of C-terminal extension of Group II. A cDNA fragment encoding a Group II phytosystatin, SiCYS was previously obtained from sesame seeds.

**Results:**

SiCYS as well as its two structural domains, N-terminal and C-terminal domains (SiCYS-N and SiCYS-C), was expressed in *Escherichia coli*. The recombinant SiCYS and SiCYS-N showed inhibitory activity against papain. The *K* i values of SiCYS and SiCYS-N were ~1.9 ×10^-8^ M and ~7.9 ×10^-8^ M, respectively. All the three recombinants possessed comparable ability to inhibit spore germination of *Trichoderma reesei*, *Aspergillus sydowii*, and *Helminthosporium sesamum*. Similar protein profile including proteases in germinating seeds was found in proteins purified by the SiCYS, SiCYS-N or SiCYS-C coupling affinity column.

**Conclusion:**

SiCYS exhibited more effective papain-inhibitory activity than SiCYS-N; while SiCYS-C had almost no inhibitory activity. All displayed similar antifungal activities indicating that there is no correlation between antifungal and papain-inhibitory activities. Structural and sequence analyses suggest that the C-terminal domain of SiCYS may be originated from gene duplication to enhance its inhibitory activity.

**Electronic supplementary material:**

The online version of this article (doi:10.1186/1999-3110-55-18) contains supplementary material, which is available to authorized users.

## Background

Cystatins are a family of proteinaceous inhibitors for papain-like cysteine proteases. They act as pseudo-substrates to enter the active-site cleft of proteases, and subsequently inhibit the proteolytic activity (Brown and Dziegielewska 1997). Cystatins initially identified from mammalian tissue are widely distributed across the animal and plant kingdoms. Three conserved motifs were found in the cystatins of animal and plant origins, a G residue near the N-terminus, a central QXVXG motif in the first hairpin loop, and a W residue in the second hairpin loop near the C-terminus. These three conserved regions within cystatin domain could form a tripartite wedge, which directly interacts with cysteine protease to cause the inhibition (Turk et al. 1997; Nagata et al. 2000). Plant cystatins, characterized as no disulfide bond and referred as phytocystatins, contain a consensus sequence [LVI]-[AGT]-[RKE]-[FY]-[AS]-[VI]-X-[EDQV]-[HYFQ]-N at N-terminal (Nt) region of an α-helix, which is absent in animal cystatins (Margis et al. 1998). The typical structure elements of phytocystatins include four β sheets linked to an α-helix in the Nt region and two hairpin loops (Nagata et al. 2000; Arai et al. 2002).

According to the molecular weight, phytocystatins are classified into three distinct groups. Group I phytocystatins have similar molecular weight, ~12 kDa as its animal homolog, chicken egg white cystatin. They share high homology with the Nt domain of the group II phytocystatins containing an additional C-terminal (Ct) extension of ~10 kDa with a conserved SNSL motif, which may be involved in the inhibition of legumin-like proteins (Martinez et al. 2007). Group III, plant multicystatins were found in potato and tomato, comprising eight tandem cystatin domains with a high molecular weight of ~85 kDa (Waldron et al. 1993; Wu and Haard 2000). A variety of phytocystatins from different groups were detected to have the inhibitory activity toward papain. After potato multicystatins was digested with trypsin, the obtained intact potato cystatin domains also showed the inhibitory activity (Nissen et al. 2009).

The physiological roles of phytocystatins are diverse, including the control of programmed cell death, storage protein turnover, plant development and growth (Solomon et al. 1999; Corre-Menguy et al. 2002; Martinez et al. 2009). Multiple seed phytocystatins were identified such as seeds from rice, sesame, and soybean (Arai et al. 2002; Shyu et al. 2004; Misaka et al. 1996). Oryzacystatin-I and -II belonging to group I were synthesized during the maturation of rice seed and decomposed as soon as germination started (Kondo et al. 1990; Abe et al. 1991). In addition, their inhibitory activities toward cysteine proteinases as well as their mRNA expression patterns during the seed maturation were distinct from each other. In transgenic *Arabidopsis*, the constitutive over-expression of BrCYS1 (a group II phytocystatin from *Brassica rapa* seeds) retarded germination and seedling growth; whereas the opposite effect was observed as BrCYS1 expression was suppressed (Hong et al. 2007). Thus, seed phytocystatins play unique roles in the control of seed germination and seedling growth probably through the regulation of different cysteine proteinases. Nevertheless, the precise mechanism is still not clear.

Phytocystatins are also involved in plant defense, as evidenced by their inhibitory ability against insects gut proteases (Aguiar et al. 2006), induction at protein or mRNA levels following wounding or methyl-jasmonate treatments (Botella et al. 1996), and the increased resistance to insects and bacterial phytopathogens in transgenic plants over-expressing a phytocystatin CeCPI from taro (Senthilkumar et al. 2010). Moreover, the ability to suppress the fungal growth has been detected *in vitro* in phytocystatins of sugarcane, strawberry, winter wheat, and taro (Soares-Costa et al. 2002; Martinez et al. 2005; Christova et al. 2006; Wang et al. 2008), though their antifungal mechanism is unknown.

Previously, a cDNA fragment encoding a sesame seed phytocystatin (SiCYS) was obtained (Shyu et al. 2004). SiCYS comprising 199 amino acid residues was predicted to have no N-terminal signal peptide and no disulfide bind with a molecular weight of 22 kDa. It belongs to group II, and is composed of an Nt domain of 88 residues and a Ct domain of 111 residues. The recombinant SiCYS over-expressed in *Escherichia coli* showed effective inhibitor activities against papain with *K* i value of ~10^-8^ M similar to other known phytocystatins with *K* i in a range of 10^-6^-10^-8^ M. To gain insights into the functions of the two domains in SiCYS, the full-length SiCYS, its Nt domain (SiCYS-N) and Ct domain (SiCYS-C) were produced in *Escherichia coli* in the present study. These recombinants were detected for their ability to inhibit papain activity as well as to arrest fungal growth. To further explore the physiological roles of SiCYS, particularly its Ct extension, the protein profile and proteases in germinating seeds was preliminarily examined.

## Methods

### Protein preparation of sesame seeds

Mature and fresh maturing sesame (*Sesamun indicum* L.) seeds were grown in the Crops Improvement Department, Taiwan District Agricultural Research and Extension Station. For germination, mature seeds were imbibed in water at 27°C for 3 days. To prepare the total extracted proteins, the seeds were grounded in a mortar with liquid nitrogen and extracted with a buffer containing 0.6 M sucrose and 0.01 M sodium phosphate, pH 7.5. The homogenate was filtrated through cheesecloth, followed by centrifugation at 4000 rpm for 20 min. The proteins in the supernatant were collected for further studies.

### Construction and over-expression of recombinant SiCYS, SiCYS-N and SiCYS-C in *E. coli*

For over-expression of non-fusion SiCYS in *E. coli*, the recombinant plasmid pET-28a containing *SiCYS* was obtained from our previous work (Shyu et al. 2004). In this study, two sets of primers with the restriction enzymes *Hind* III and *Nde* I, (5′-GGCAGCCATATGGCTACTCTAGGGGGC-3′ and 5′-GCAAGCTTAATGTTTGAATTCTTGTAGTTG-3′; 5′-GGCAGCCATATGGTTCGAGATGTTCCTTCTTTTAC-3′ and 5′-GCAAGCTTAAGAGTGATCAAGATCCACC-3′) were designed and used to amplify the DNA fragments encoding the N- and C-terminal regions of SiCYS, SiCYS-N and SiCYS-C by PCR, respectively. After restriction digestion, each amplified DNA fragment was separately ligated into an expression vector, pET28a (Novagen) and then transformed into *E. coli* DH10B. For over-expression of His-tagged SiCYS-C, the expression vector, pET-29a (Novagen) was used. The nucleotide sequence of the insert was confirmed by sequencing. Following transformation into *E. coli* BL21(DE3), the over-expression of recombinant protein was induced with 1 mM isopropyl-*β*-D-thiogalactopyranoside (IPTG). After IPTG induction for 3 hours at 37°C, the *E. coli* cells were harvested and lysed by sonication in a 10 mM phosphate buffer (pH 8.0). They were then fractionated into soluble and precipitated fractions by centrifugation, followed by protein analyses and purification.

### Preparation of affinity column and purification of recombinant proteins

For nonfusion recombinant proteins, papain coupled column was prepared, described in our previous work (Shyu et al. 2004). Papain as the binding ligand was coupled to CNBr-activated Sepharose 4B (Amersham Biosciences) following the manufacturer’s instruction. Then, the nonfusion soluble fraction of cell lysate were stirred overnight with the papain-Sepharose 4B previously equilibrated with 50 mM sodium phosphate buffer, pH 6.5 containing 0.5 M NaCl and 0.1% Brij 35, and washed with 50 mM sodium phosphate buffer, pH 6.5 containing 0.5 M NaCl and 10% (v/v) glycerol, the proteins were eluted with 50 mM K_3_PO_4_, pH 11.5 containing 0.5 M NaCl and 10% glycerol. The eluent were adjusted to pH 7.4 with 5 M sodium formate buffer, pH 3. A Ni^2+^-NTA column (Novagen) was applied to purify the proteins with poly-histidine tail such as His-tagged SiCYS-C. Following the manufacturer’s protocol, His-tagged SiCYS-C was eluted from the column with a 50 mM sodium phosphate buffer, pH 7 containing 0.3 M NaCl and 150 mM imidazole. The Amicon Ultra-15 column (Millipore) was applied to concentrate the proteins in a PBS buffer, pH 7.5 using a modified manufacturer’s protocol. Finally, proteins concentration was determined by BCA Protein Assay Kit (Pierce).

### Papain inhibition assay

Protease inhibitory assay was performed using papain as the target enzyme and *N*-benzoyl-L-arginine-2-naphthylamide (BANA) as the substrate (BANA and papain were purchased from Sigma-Aldrich. Various amounts of recombinant proteins were incubated with 10 μg of papain (2.9 U/mg) in a 250-μl assay solution containing 0.1 M sodium phosphate at pH 6.0, 1 mM EDTA, 2 mM β-mercaptoethanol for 5 min at 37°C. Then, the reaction was initiated by adding 0.1 mL of 1 mM BANA for 10 min at 37°C, and terminated by adding 0.5 ml of 2% HCl/ethanol for 10 min. The color was developed after the addition of 0.5 ml of 0.06% *p*-dimethylamino-cinnamaldehyde in ethanol for 15 min, and the absorbance of mixture was measured at 540 nm. Papain residual activity (%) was calculated by the equation, (A*/A) × 100%, where A and A* represented the ΔOD_540_ in the absence and presence of the recombinant protein compared to the zero control, respectively. The inhibitory activity was expressed as the percentage decrease in papain residual activity as the equation, 1-[(A*/A) × 100%]. The *K* i value was determined by plots (1/v versus [I]) (Dixon 1953), where v represented protease residual activity and [I] was the concentration of recombinant protein.

### Antifungal and antibacterial assay

The purified recombinant proteins were applied to examine the growth inhibition of fungi, including *Trichoderma reesei*, *Aspergillus sydowii*, *Helminthosporium sesamum*. The fungi were cultured in potato dextrose agar for 1–2 weeks. Approximately 10^4^ spores of each strain were incubated in 3 ml of potato dextrose broth with or without the recombinant protein at 28°C for 48 h under continuous shaking (180 rpm). Inhibition of spore germination and mycelial growth was observed under a light microscope. For antibacterial activity assay, sterile filter discs papers were placed on Muller-Hinton plates with different strain of bacteria including Gram-positive bacteria (*Streptococcus pneumonia*, *Staphylococcus aureus* and *Bacillus subtilis*) and Gram-negative bacteria (*Pseudomonas aeruginosa*, *Burkholderia cepacia*, and *Pseudomonas putida*). After addition of recombinant protein discs papers, the plates were incubated at 37°C overnight and the clear zone diameter was visually monitored as the inhibition of bacterial growth. The experiment was carried out at least three times independently using the same conditions.

### Pull-down of endogenous proteins by affinity columns

According to the procedure for the preparation of papain-coupled affinity column, the purified recombinant SiCYS, SiCYS-N and His-tagged SiCYS-C were used as ligands to prepare affinity columns, respectively. The columns were further applied for the isolation of endogenous proteins in the mature or germinated seeds, which have interaction with SiCYS, SiCYS-N or His-tagged SiCYS-C. The protein supernatant extracted from seeds was incubated with recombinant protein-coupled resins overnight at 4°C, followed by package of the column. Then, the column was washed three times with 50 mM sodium phosphate buffer, pH 6.5 containing 0.5 M NaCl and 10% (v/v) glycerol. The proteins that bound to the recombinant protein-column was elute with 50 mM K_3_PO_4_, pH 11.5 containing 0.5 M NaCl and 10% glycerol. Subsequently, the eluent containing the affinity-purified endogenous proteins/cysteine proteases was adjusted to pH 7.4 and concentrated with Amicon Ultra-15 filter devices.

### Zymographic analyses of protease activity

The affinity-purified endogenous proteases were analyzed in a 0.1% gelatin containing SDS-PAGE gel for zymographic assay of proteases activity. After electrophoresis, the renaturation step was performed by washing the gel twice with a 10 mM Tris–HCl buffer at pH 7.5 containing 2.5% (v/v) Triton X-100 with gentle agitation for 30 min at room temperature. After renaturation, the gel was incubated in a protease activation buffer (0.1 M phosphate buffer at pH 6.0 containing 2 mM cysteine and 1 mM EDTA) at 37°C for 1 h. The proteolytic reaction was terminated by transferring the gel to a staining solution containing Coomassie Brilliant Blue R250. After gel destaining, the proteolysis activity was appeared as clear zones on the gel resulting from the digestion of gelatin by active proteases.

### Sequence analysis and molecular modeling

The structural homologous modeling of SiCYS-N and SiCYS-C was built using the NMR structure of Orzyacystatin I (PDB code, 1EQK) (Nagata et al., 2000) as template, and conducted by the automated SWISS-MODEL program (Guex and Peitsch, 1997; Arnold et al. 2006; Schwede et al. 2003). Sequence alignment was performed using the ClustalW algorithm as part of Megalign (DNASTAR Inc., Madison, WI, USA) and modified.

## Results

### Over-expression of various regions of SiCYS in *E. coli* and purification of recombinant proteins

**Figure 1:**
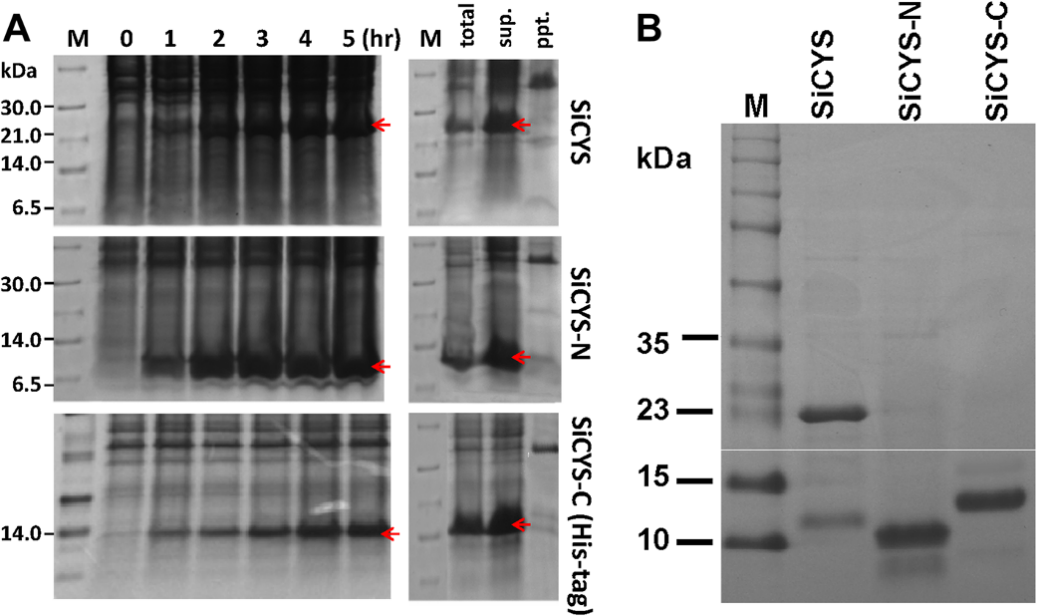
**SDS-PAGE analysis of sesame phytocystatins over-expressed in** ***E. coli*** **. (A)** Before or after IPTG induction for 1–5 hours (hr), the total proteins were extracted from ***E. coli*** cells carrying recombinant vectors with DNA fragment, *SiCYS*, *SiCYS-N* or *SiCYS-C*, followed by SDS-PAGE analyses. The total proteins (total) extracted from ***E. coli*** cells after 3 hr IPTG induction were fractionated into precipitate (ppt.) and supernatant (sup.), and then resolved by SDS-PAGE. The protein bands of over-expressed recombinants on SDS-PAGE with the expected molecular weight, 22 kDa for SiCYS, 10 kDa for SiCYS-N or 14 kDa for His-tagged SiCYS-C were marked by red arrows. **(B)** The non-fusion recombinants, SiCYS and SiCYS-N were purified via papain-coupled affinity chromatography; while His-tagged SiCYS-C was purified via nickel affinity chromatography. Lane 1, 2 and 3 were the purified recombinant SiCYS, SiCYS-N and His-tagged SiCYS-C on SDS-PAGE analysis, respectively. Lane M represent as protein marker.

Primers were designed to amplify the *SiCYS* cDNA fragments coding for the SiCYS, SiCYS-N and SiCYS-C. SiCYS-N and SiCYS-C represent 1–88 and 89–199 a.a. of SiCYS, respectively. Thus, SiCYS comprises 199 amino acid residues with a predicted molecular weight of 22 kDa, SiCYS-N comprises 88 amino acids with a molecular weight of 10 kDa, and SiCYS-C comprising 111 amino acids has an expected 12 kDa. The DNA fragments were separately cloned into *Hind* III and *Nde* I sites of pET-29a, a non-fusion expression vector, respectively. The recombinant plasmids were then transformed into *E. coli* BL21 (DE3) for protein expression separately. Compared to without IPTG induction, the recombinant non-fusion SiCYS, SiCYS-N were successfully over-expressed as soluble proteins in *E. coli* (Figure 1A), as well as non-fusion SiCYS-C (data not shown)*.*

The recombinant SiCYS and SiCYS-N could be further purified by the affinity column coupled with papain; in contrast, SiCYS-C was failed to be purified by the affinity column. It might be due to the lack of the three conserved regions essential for the binding to papain in SiCYS-C. To improve and simplify the purification steps, SiCYS-C was then constructed into pET-28a, a His-tagged fusion expression vector. After IPTG induction, His-tagged fusion SiCYS-C was over-expressed as a soluble protein in *E. coli* (Figure 1A) and purified via a His-tag affinity column. Figure 1B showed that the purified recombinant proteins, SiCYS, SiCYS-N and SiCYS-C (His-tagged) with the expected, 22, 10 and 14 kDa in SDS-PAGE, respectively.

### Papain inhibitory activity of recombinant proteins

**Figure 2:**
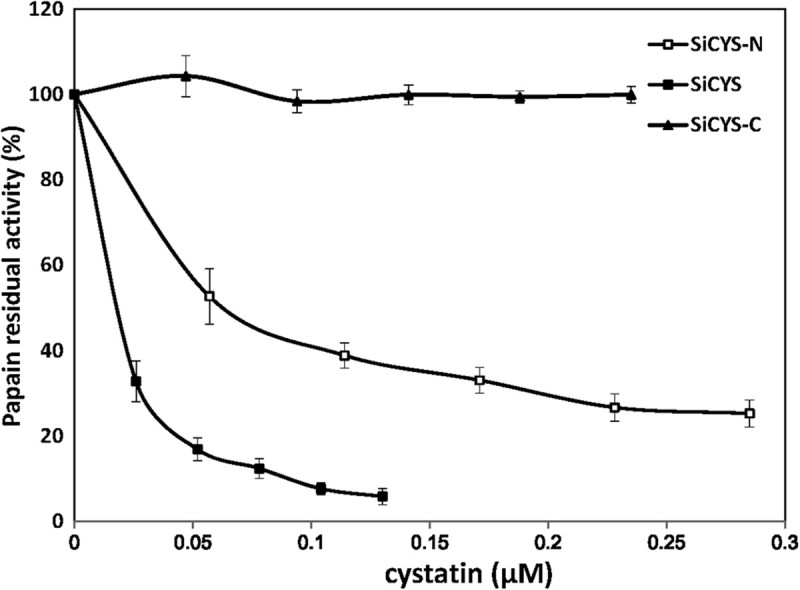
**Inhibition of papain activity by recombinant sesame phytocystatins.** Papain was mixed with various amounts of recombinant SiCYS, SiCYS-N and His-tagged SiCYS-C, respectively. The residual hydrolytic activity was determined and present as percentage of total activity of papain. The final concentration of papain was 1.24 μM in a 350 μl reaction. The added recombinants were depicted as the concentration of cystatin, μM as well. The assay was performed three independent times and presented as mean ± S.E.

SiCYS, SiCYS-N and SiCYS-C were subjected to the inhibitory activity assay again papain (Figure 2). SiCYS-C exhibited extremely weak inhibitory activity in comparison with the control without any protein. It was in accordance with the fact that SiCYS-C could not efficiently bind to the affinity column coupled with papain. SiCYS and SiCYS-N could inhibit papain activity, and their *K* i values were ~1.9 ×10^-8^ M and ~7.9×10^-8^ M, respectively.

### Anti-microbial activity of recombinant proteins

**Figure 3:**
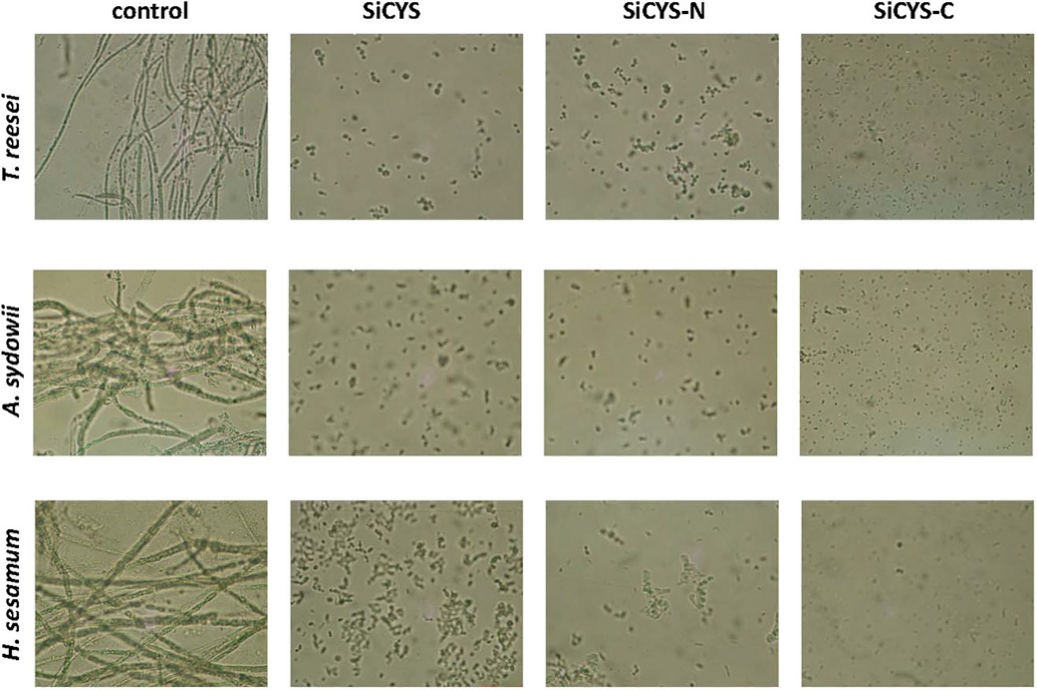
**Growth arrests of fungi by recombinant sesame phytocystatins.** The inhibition of *T. reesei*, *A. sydowii* and *H. sesamum* spore germination by SiCYS, SiCYS-N or His-tagged SiCYS-C at 45 μg/ml of concentration were observed by microscopy comparing to the control, without any recombinant.

Phytocystatins have gained attention as they protect plants from the invasion of fungi with unclear mechanism. To examine the effects of SiCYS and its two domains on antifungal activity, the recombinant SiCYS, SiCYS-N and SiCYS-C were subjected to the growth inhibition assay of two severe pathogenic fungi, *A. sydowii* and *H. sesamum*, as well as *T. reesei. H. sesamum* causing an aerial stem rot disease in sesame plants. Soil borne *A. sydowii* contaminates food and is occasionally pathogenic to humans. The soil borne *T. reesei* is generally regared as a non-pathogenic fungus, and is well known for the capacity to secrete large amounts of cellulolytic enzymes for industrial application. Ten-100 μg/ml of recombinant proteins were initially examined for anti-fungal activity assay (data not shown). Around 45 μg/ml of each recombinant almost completely inhibited the spore germination and the mycelial growth of *A. sydowii*, *H. sesamum* and *T. reesei*, after incubation at 25°C for 48 h. The results were corroborated by microscopic observations, as shown in Figure 3. Moreover, SiCYS, SiCYS-N or SiCYS-C had no effect on the proliferation of various bacteria (data not shown).

#### SDS-PAGE and zymographic analyses of proteins binding to affinity columns

**Figure 4:**
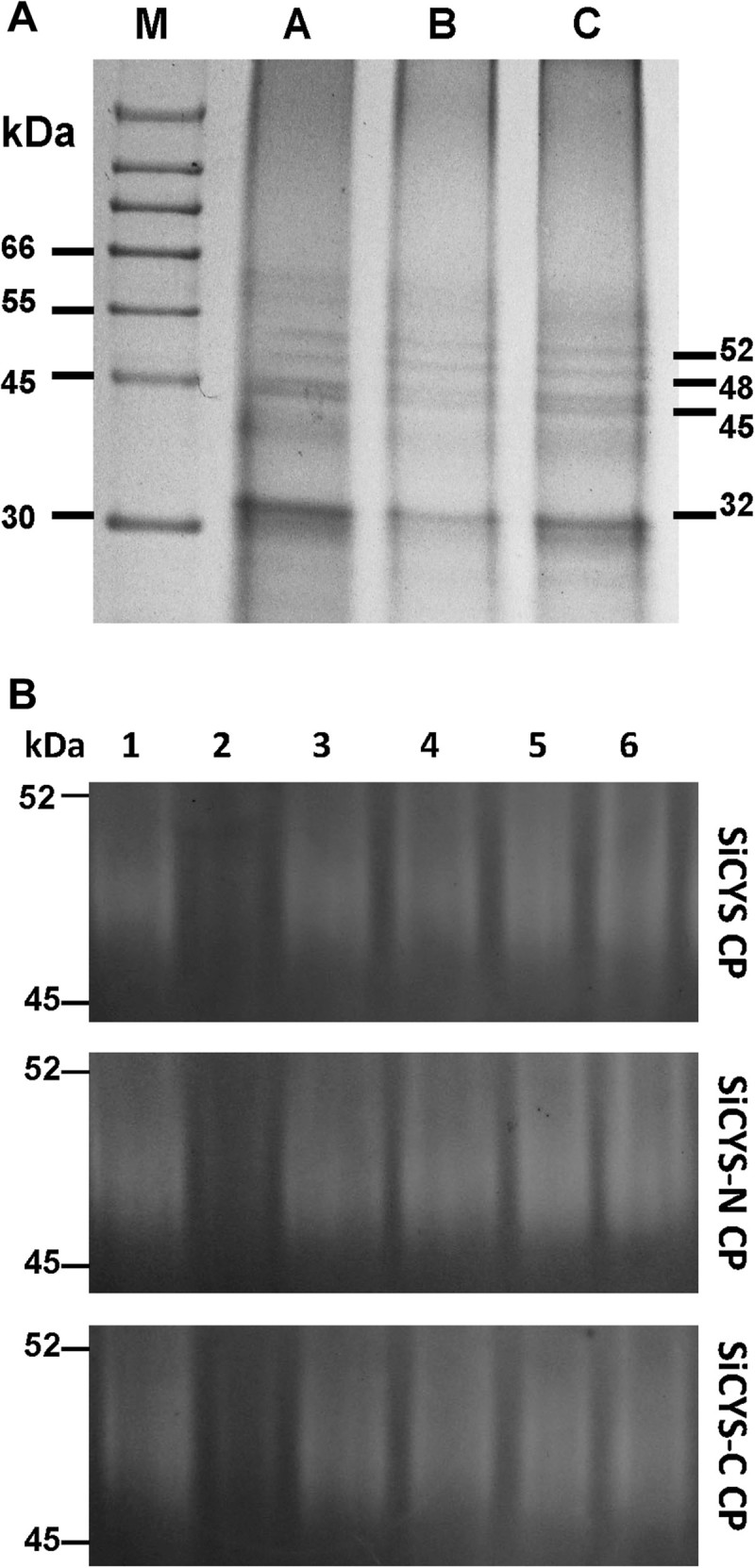
**SDS-PAGE and zymographic analyses of germinated sesame proteins elute from affinity columns. (A)** The proteins extracted from the germinated sesame were collected after the purification with affinity columns, followed by SDS-PAGE analyses. Lane A, B and C were the pull-down proteins/extracted proteins via SiCYS, SiCYS-N and SiCYS-C coupled affinity columns; namely SiCYS CP, SiCYS-N CP and SiCYS-C CP, respectively. The protein bands with molecular weight of ~32, ~45, ~48 and ~52 kDa in SDS-PAGE were marked. **(B)** SiCYS CP, SiCYS-N CP and SiCYS-C CP were added with or without protease inhibitors, followed by zymographic analyses of protease activity. Lane 2 was added with 20 mM of PMSF; lane 3, 250 μM of E64; lane 4, 5 mM of iodoacetamide; lane 5, 5 mM of *N*-ethylmaleimide, lane 6, 100 μM of pepstatin A; lane 7, 25 mM of EDTA; while no any inhibitor was added in lane 1. The location of ~45 and ~52 kDa on gel was marked as well.

It has been suggested that SiCYS plays a role in the control of seed development or germination by regulating the activities of endogenous cysteine protease/target proteins (Shyu et al. 2004). To explore potential endogenous protease/target proteins, affinity columns coupled with SiCYS, SiCYS-N and SiCYS-C were prepared, respectively. The proteins extracted from the germinated sesame were applied onto the SiCYS, SiCYS-N or SiCYS-C coupled affinity columns, respectively and then eluted for analyses. All the pull-down endogenous proteins, termed SiCYS CP, SiCYS-N CP, or SiCYS-C CP resolved by SDS-PAGE, displayed a similar pattern, particularly, the protein bands with molecular weight of approximately 32, 45, 48 and 52 kDa (Figure 4A). Following the treatment with various protease inhibitors including PMSF (for Ser protease), E64, iodoacetamide and N-ethylmaleimide (for Cys protease), pepstatin A (for Asp protease) or EDTA (for metallo-protease) (García-Carreño 1992), SiCYS CP, SiCYS-N CP and SiCYS-C CP were subjected for zymographic analyses of protease activity (Figure 4B). Only PMSF could almost completely inhibit the protease activity, suggesting that putative serine proteases from the germinated sesame were mainly associated with SiCYS, SiCYS-N or SiCYS-C.

### Structural analysis of SiCYS-N and SiCYS-C

**Figure 5:**
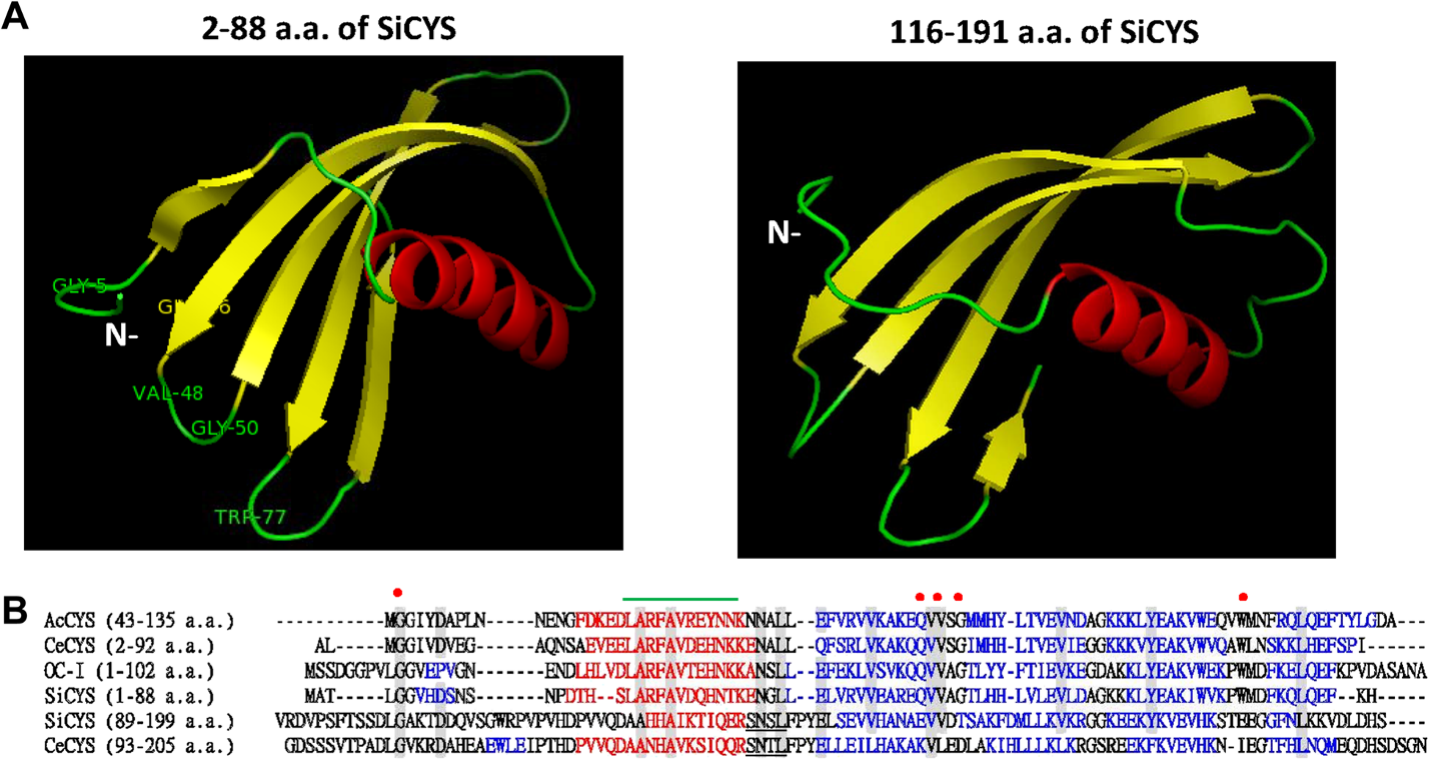
**The predicted structures of SiCYS-N and SiCYS-C, and their sequence alignment with other phytocystatins. (A)** The main three dimensional structure of SiCYS-N (2–88 a.a of SiCYS) and SiCYS-C (116–191 a.a. of SiCYS) shown in cartoon diagram was predicted by homology modeling using Orzyacystatin I (PDB: 1EQK) as template. The secondary structure elements corresponding to α-helices and β-sheets were shown in red and yellow. Three conserved motifs (G^5^, Q^46^XV^48^XG^50^, W^77^) were labeled. **(B)** SiCYS-N (1–88 a.a. of SiCYS) and SiCYS-C (89–199 a.a. of SiCYS) was aligned with Orzyacystatin I (term OC-I), AcCYS and CeCPI. The structures of Orzyacystatin I, AcCYS and CeCPI (2–92 a.a.) but not CeCPI (93–205 a.a.) were currently available in PDB. Amino acid residues identical at least five out of six aligned sequences were boxed in grey. Three conserved motifs were signed in red dot. A specific consensus sequence of phytocystatins, LARFAVXQHN, was marked in green upper line; while SN(S/T)L motif found in Ct extension marked in under line. The sequence corresponding to α-helice and β-sheets were signed in red and blue.

SiCYS-N and SiCYS-C are the polypeptides with 1–88 and 89–199 a.a. of SiCYS, respectively. By using the structure of rice Orzyacystatin I (a group I phytocystatin, PDB, 1EQK) as a template (Nagata et al. 2000), the main three dimensional structures of SiCYS-N and SiCYS-C were constructed, respectively (Figure 5A). SiCYS-N shares 53.93% identity in 87 a.a. with Orzyacystatin I; while SiCYS-C has 23.81% identity in 89 a.a. with Orzyacystatin I. SiCYS-N and SiCYS-C share 18.8% identity to each other, and their predicted three dimensional structures are alike, as revealed in Figure 5A. SiCYS-N is mainly composed of one main α helice and five antiparallel β sheets; while SiCYS-C is composed of one main α helice followed by four antiparallel β sheets. The taro CeCPI belongs to the group II containing Ct extension. The X-ray structure of its Nt region (2–92 a.a.) was obtainable, PDB: 3IMA (Chu et al. 2011); however, the structure of Ct extension was not solved yet. The NMR structures of pineapple AcCYS, a group I phytocystatin with an extra Nt extension was also available (PDB, 2L4V) as well (Irene et al. 2012). By using CeCPI (2–91 a.a.) and AcCYS (43–135 a.a.) as templates, the structures of either SiCYS-N or SiCYS-C showed similarity as those using Orzyacystatin I as templates (data not shown).

Figure 5B showed the sequence alignment of SiCYS-N (1–88 a.a. of SiCYS) and SiCYS-C (89–199 a.a. of SiCYS) with Orzyacystatin I, CeCPI (2–92 and 93–205 a.a.) and AcCYS (43–135 a.a.). Three conservative regions, proposed to interact with cysteine protease, were found in SiCYS-N, Orzyacystatin I, CeCPI, and AcCYS: a G residue near the N-terminus, a central QXVXG motif in the first hairpin loop, and a W residue in the second hairpin loop near the C-terminus putatively forming a tripartite wedge. SiCYS-C contains the SN(S/T)L motif as found in the Ct extension of other group II phytocystatins, including CeCPI.

## Discussion and conclusions

In this study, both SiCYS and SiCYS-N possessed inhibitory activities against papain; while SiCYS-N exhibited less ability to inhibit papain activity compared to SiCYS. Similarly, the recombinants removing Ct region from several group II phytocystatins, such as soybean soycystatin and taro CeCPI, showed comparable or less inhibitory activity against papain as their full-length one (Misaka et al. 1996; Wang et al. 2008). Almost no inhibitory activity against papain was associated with SiCYS-C, neither for the recombinant Ct domain of CeCPI (Wang et al. 2008). The weak papain activation property of Ct domain of CeCPI was addressed based on the study of inhibitory kinetics with the full-length, Nt and Ct domain of CeCPI (Wang et al. 2008). However, no apparent papain activation was found with SiCYS-C in this study. In gel zymographic analyses of papain activity also showed that Ct domain of CeCPI could enhance papain activity, suggesting that Ct domain of CeCPI alone may play a role to alter papain conformation (Wang et al. 2008). A regulatory effect of Ct extension on the function of CeCPI was thus proposed together with the results, a shift in the inhibitory pattern from competitive inhibition of its Nt domain alone to mixed type inhibition of its full-length one (Wang et al. 2008).

SiCYS showed a better inhibitory activity of papain than SiCYS-N. The average *K* i value of SiCYS, ~1.9 ×10^-8^ M was less than that of SiCYS-N, ~7.9×10^-8^ M. Though the *K* i value of SiCYS was less in this study than the previous work, ~2.77 ×10^-8^ M (Shyu et al. 2004), both *K* i values are consistent as considering the standard error. Ct extension in SiCYS/Group II phytocystatins presumably did not obstruct the binding of its Nt region to papain, but improves the inhibitory activity against papain bases on the studies with CeCPI (Wang et al. 2008) and SiCYS here. SiCYS-C, Ct domain of SiCYS could not efficiently bind to papain coupled affinity column and had no apparent effect on proteolysis of papain. Ct domain of CeCPI could enhance papain activity (Wang et al. 2008), and could be easily digested by papain (Chu et al. 2011). Nevertheless, how Ct extension in Group II phytocystatins affects the target protease/papain activity is still unclear, and remains to be studied.

The principal function of cystatins of plant and animal originals is usually attributed to their inhibitory activities against the exogenous and endogenous cysteine proteases. Phytocystatins have been demonstrated to be defense proteins to inhibit exogenous cysteine proteases upon the invasion of pathogens or pests, whose cysteine proteases play a role in cell replication or serve as digestive enzymes in the gut (Arai et al. 2002). Furthermore, phytocystatins were demonstrated to have antifungal activity (Soares-Costa et al. 2002; Martinez et al. 2005; Christova et al. 2006; Wang et al. 2008). SiCYS, SiCYS-N and SiCYS-C possess the ability to *in vitro* suppress the spore germination of fungi including *T. reesei*, *A. sydowii*, and *H. sesamum*, respectively. Surprisingly, SiCYS-C exerted comparable antifungal activity as SiCYS and SiCYS-N. Presumably, the antifungal activity of SiCYS is not accountable for their inhibitory activity against papain. Though SiCYS, SiCYS-N or SiCYS-C was purified to high purity by two different affinity columns, it could not be completely excluded that the antifungal effect might be also partially resulted from the trace contaminant in the purified protein.

The studies of HvCPI, a group I phytocystatin from barley, and a series of its site-directed mutants demonstrated that antifungal activity of HvCPI is not related to its inhibitory property against cysteine protease (Martinez et al. 2003). Nt domain of CeCPI was reported to be stronger than its full-length one in the inhibition of *S. rolfsii* mycelium growth; while its Ct domain showed no inhibition effect on mycelium growth (Wang et al. 2008). In short, the ability of phytocystatins to inhibit fungal growth is not associated with their inhibitory activity against cysteine proteases. The anti-fungal mechanism exerted by phytocystatins is not clear yet; presumably, the phytocystatins suppress fungal growth by altering the permeability of fungal membrane (Martinez et al. 2005). Along with its Nt and Ct domains, SiCYS could be the good candidates for further application as pest/fungicides or in transgenic plants to enhance plant resistance toward pest and pathogens.

During seed maturation, various proteins predominantly storage proteins are synthesized and subcellularly localized in protein bodies; whereas the storage proteins are degraded and remobilized during the seedling development (Müntz et al. 2001; Tan-Wilson and Wilson, 2012). In those complicated processes, endogenous proteases and protease inhibitors delicately participate and control counterpart activity. Different families of proteases coexist in plants, and cysteine proteases are regarded as the most abundant proteases responsible for degradation and mobilization of storages proteins in seeds (Grudkowska and Zagdanska, 2004;Sreenivasulu et al. 2008). SiCYS appeared in the maturing seeds around 14 days after flowering and progressively accumulated till seed maturation; it declined rapidly in germination and disappeared 2 days after imbibitions (Shyu et al. 2004).

An effort was tried to figure out the target proteins/cysteine proteases from either mature or germinated sesame by the pull-down experiments to obtain SiCYS CP, SiCYS-N CP or SiCYS-C CP using SiCYS, SiCYS-N or SiCYS-C coupled affinity columns. Similar protein profiles from germinated sesame were subsequently observed on SDS-PAGE analyses. Zymographic analyses of protease activity suggested that the putative serine proteases were the main proteases among SiCYS CP, SiCYS-N CP or SiCYS-C CP, not the expected cysteine protease. Phytocystatins are natural inhibitors of cysteine protease. Therefore, those putative serine proteases are probably not the specific target proteases of SiCYS. Moreover, no consistent results were obtained from the study with mature sesame, as well as no obvious proteases were detected by zymographic analyses under our experimental condition (data not shown). Our preliminary results suggested that there are probably only few cysteine proteases in mature or germinated sesame, and an improved method is needed in the future to identify the target cysteine proteases of SiCYS. On the other hand, the protein identification of SiCYS CP, SiCYS-N CP or SiCYS-C CP by MALDI-TOP may provide us more information about the germinating process of sesame.

Phytocystatins of Group I and Group II differ in the additional Ct domain, whose physiological significance is still not clear. Perhaps, the Ct domain is involved in its substrate specificity to proteases, the affinity to proteases, the conformation change of proteases, the protection from degradation caused by non-cysteine proteases, or allosteric regulation of inhibitory activity against proteases. Legumins, a family of Asn-specific cysteine proteases, are involved in the protein processing. HvCPI-4 and FaCPI (Group II phytocystatins from barley and strawberry) were reported to be able to inhibit papain, human legumain and barley legumain-like activity (Martinez et al. 2007). The Ct domain of HvCPI-4 could inhibit legumain activity as well (Martinez et al. 2007). Whether SiCYS or SiCYS-C possesses the legumain inhibitory activity remains to be seen.

Apart from the weak papain inhibitory activity, SiCYS-C has antifungal effect comparable to SiCYS and SiCYS-N. Moreover, two domains of cystatin-like superfamily were found with SiCYS, as the domain search by Blast analysis at NCBI. One is localized in the Nt domain with definitive function to inhibit papain-like proteases, and the other is localized in the Ct domain with almost no inhibitory activity. Sequence alignment showed that both share a certain degree of identity. The structures of Group I, Orzyacystatin I and AcCYS could be provided for homology modeling as well as Nt region of CeCPI; while no structure of Ct extension of Group II is currently available. The structures of SiCYS-N and SiCYS-C appeared alike using those structures as templates for structural prediction. Accordingly, growing studies including ours support that Ct extension may derive from gene duplication and reserve under evolutionary selection (Martinez et al. 2007; Wang et al. 2008).

## Authors’ original submitted files for images

Below are the links to the authors’ original submitted files for images. Authors’ original file for figure 1 Authors’ original file for figure 2 Authors’ original file for figure 3 Authors’ original file for figure 4 Authors’ original file for figure 5 Authors’ original file for figure 6 Authors’ original file for figure 7
