# Supplementary figures and images for: Functional characterization of the N-terminal and C-terminal domains of a sesame group II phytocystatin
Source: Bot Stud. 2014 Feb 3;55:18. doi: 10.1186/1999-3110-55-18 (PMC5432954; doi:10.1186/1999-3110-55-18)

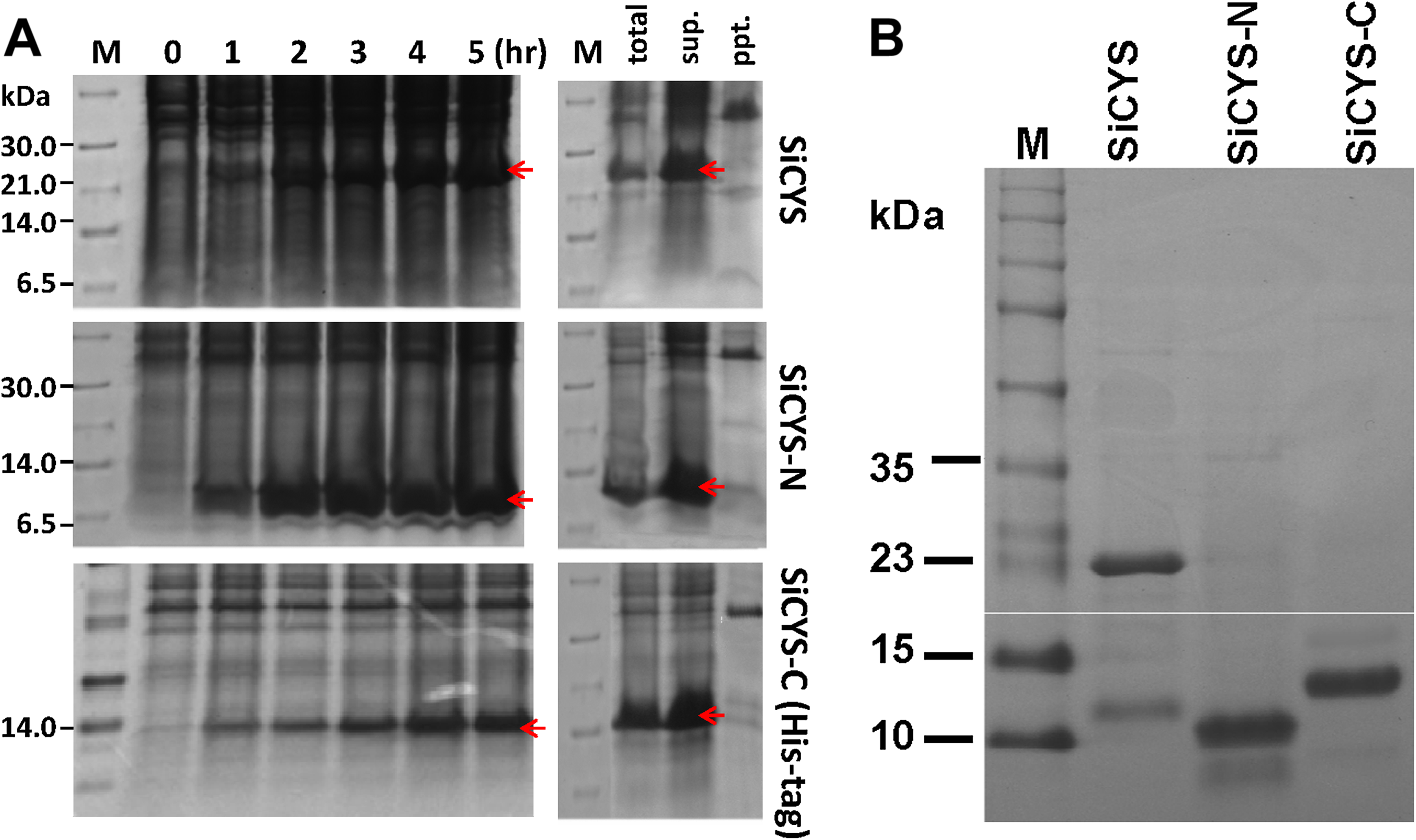

Supplement: Supplementary file 1 — Authors’ original file for figure 1 [file 40529_2013_77_MOESM1_ESM.tiff]

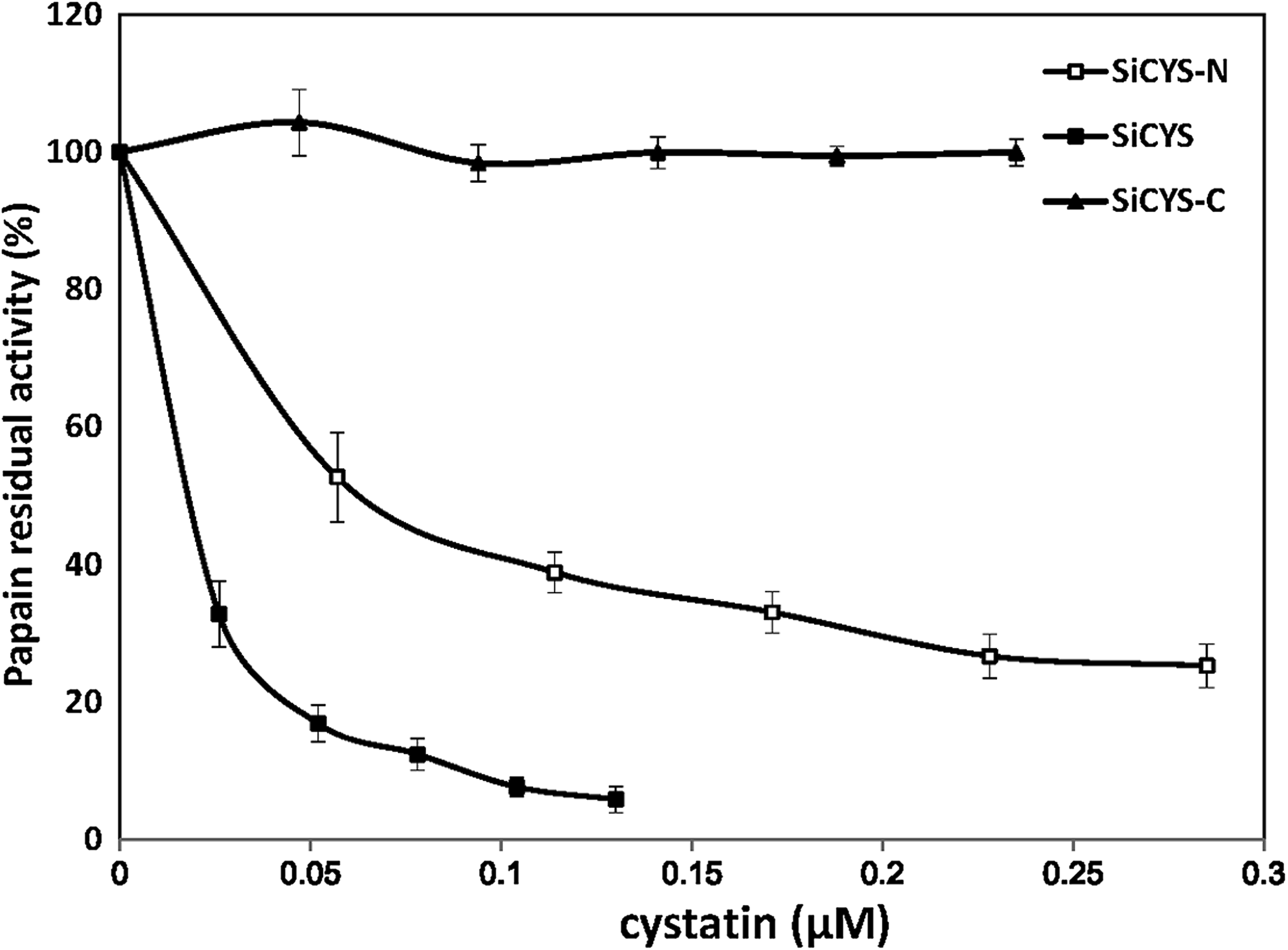

Supplement: Supplementary file 2 — Authors’ original file for figure 2 [file 40529_2013_77_MOESM2_ESM.tiff]

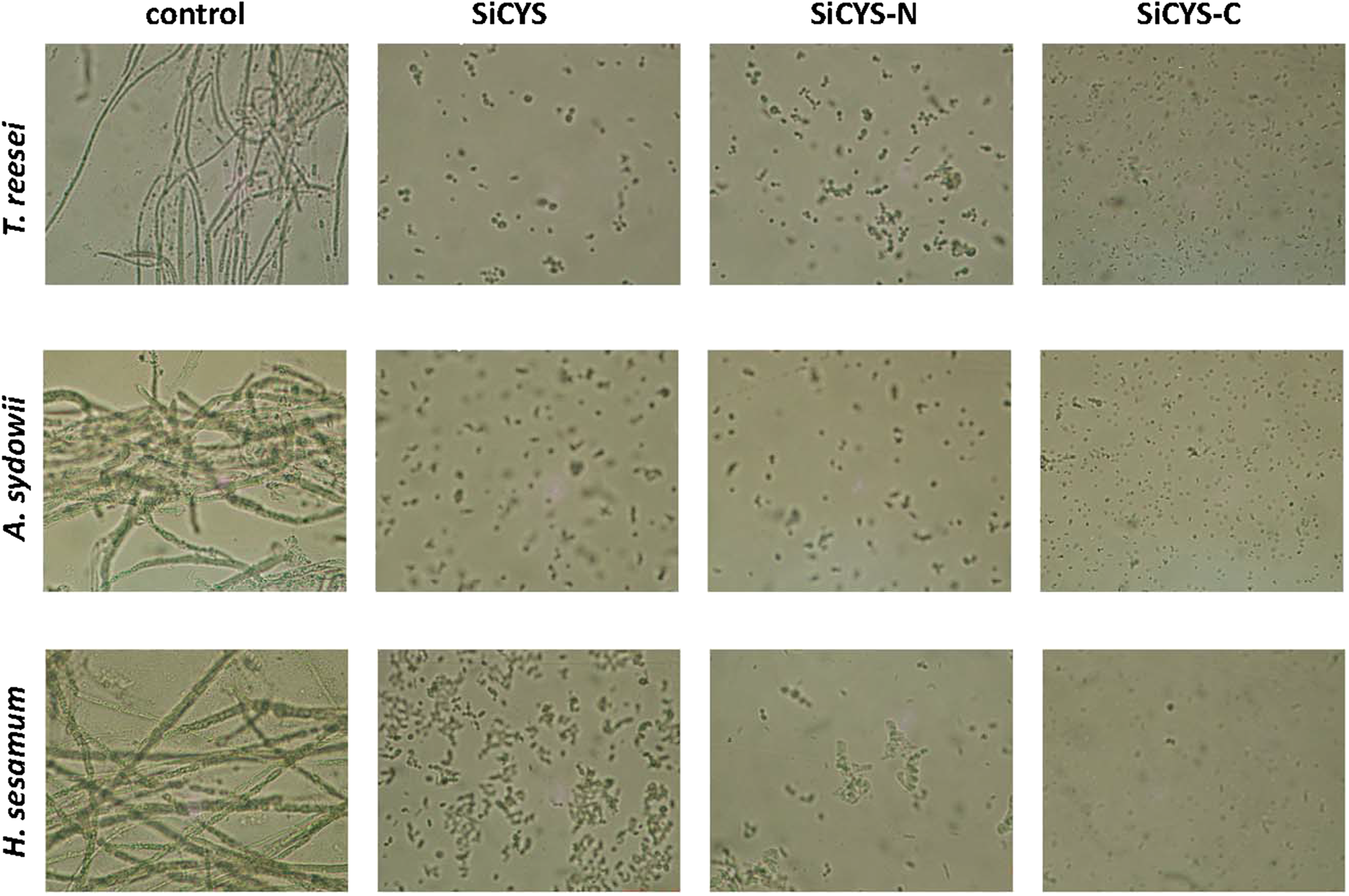

Supplement: Supplementary file 3 — Authors’ original file for figure 3 [file 40529_2013_77_MOESM3_ESM.tiff]

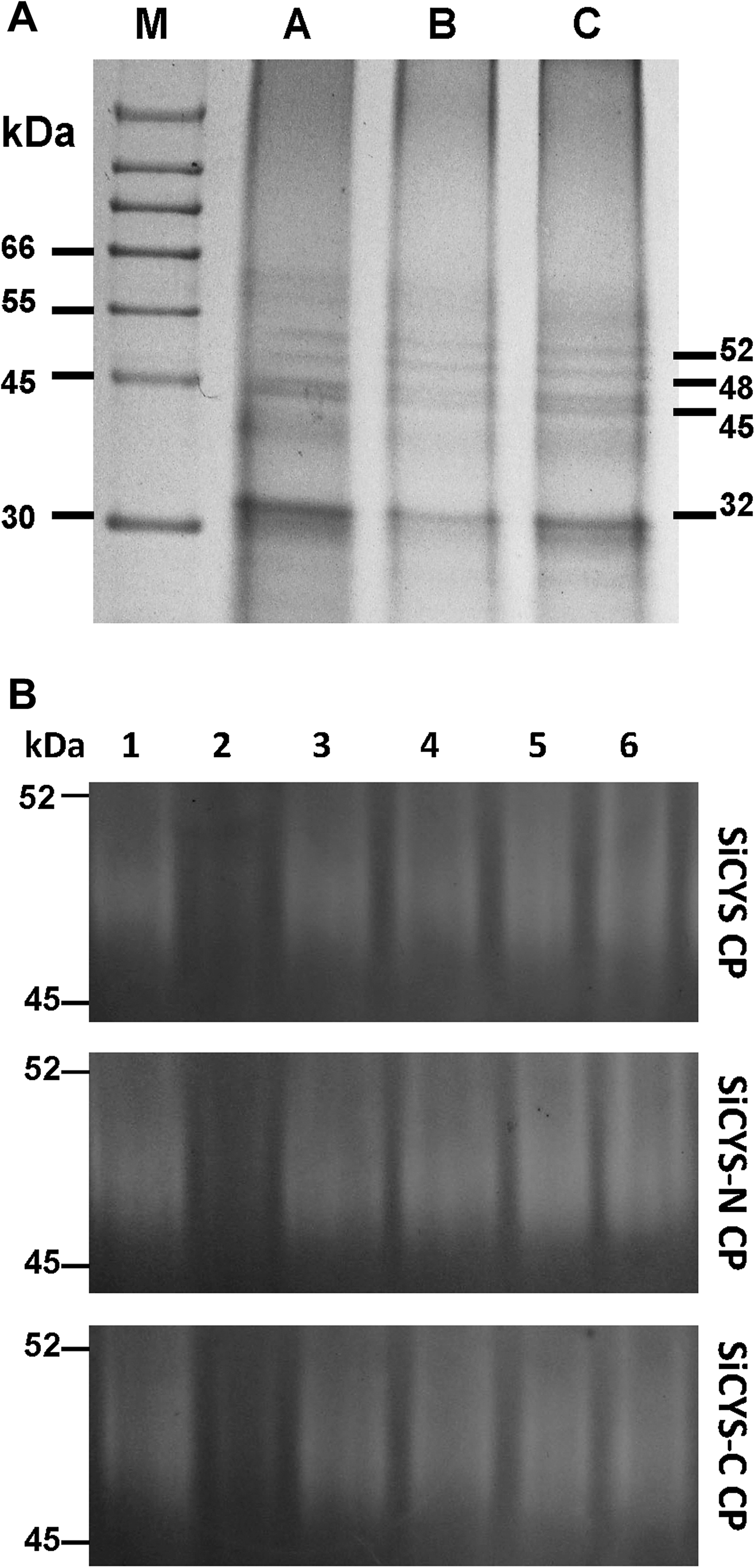

Supplement: Supplementary file 4 — Authors’ original file for figure 4 [file 40529_2013_77_MOESM4_ESM.tiff]

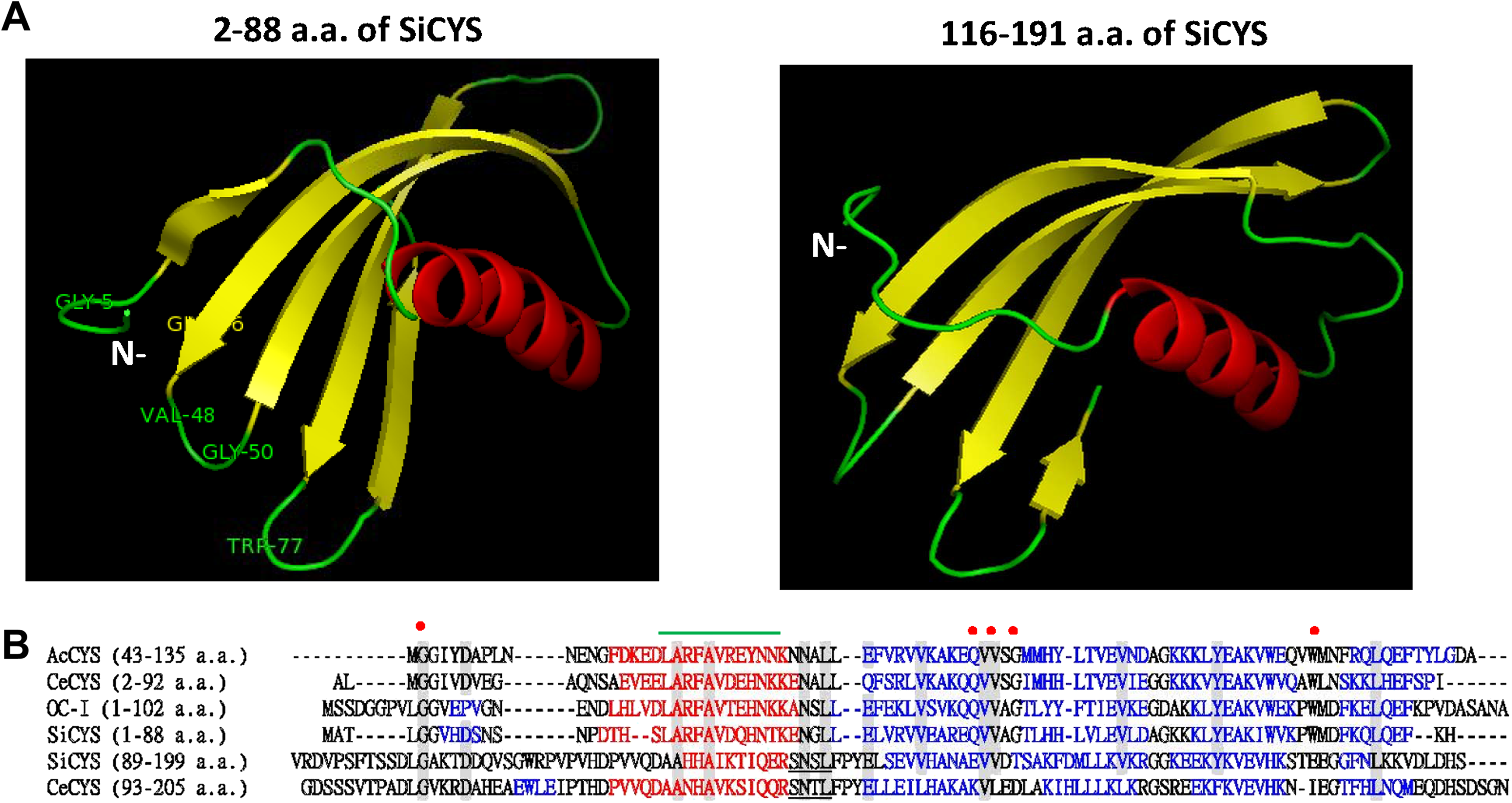

Supplement: Supplementary file 5 — Authors’ original file for figure 5 [file 40529_2013_77_MOESM5_ESM.tiff]

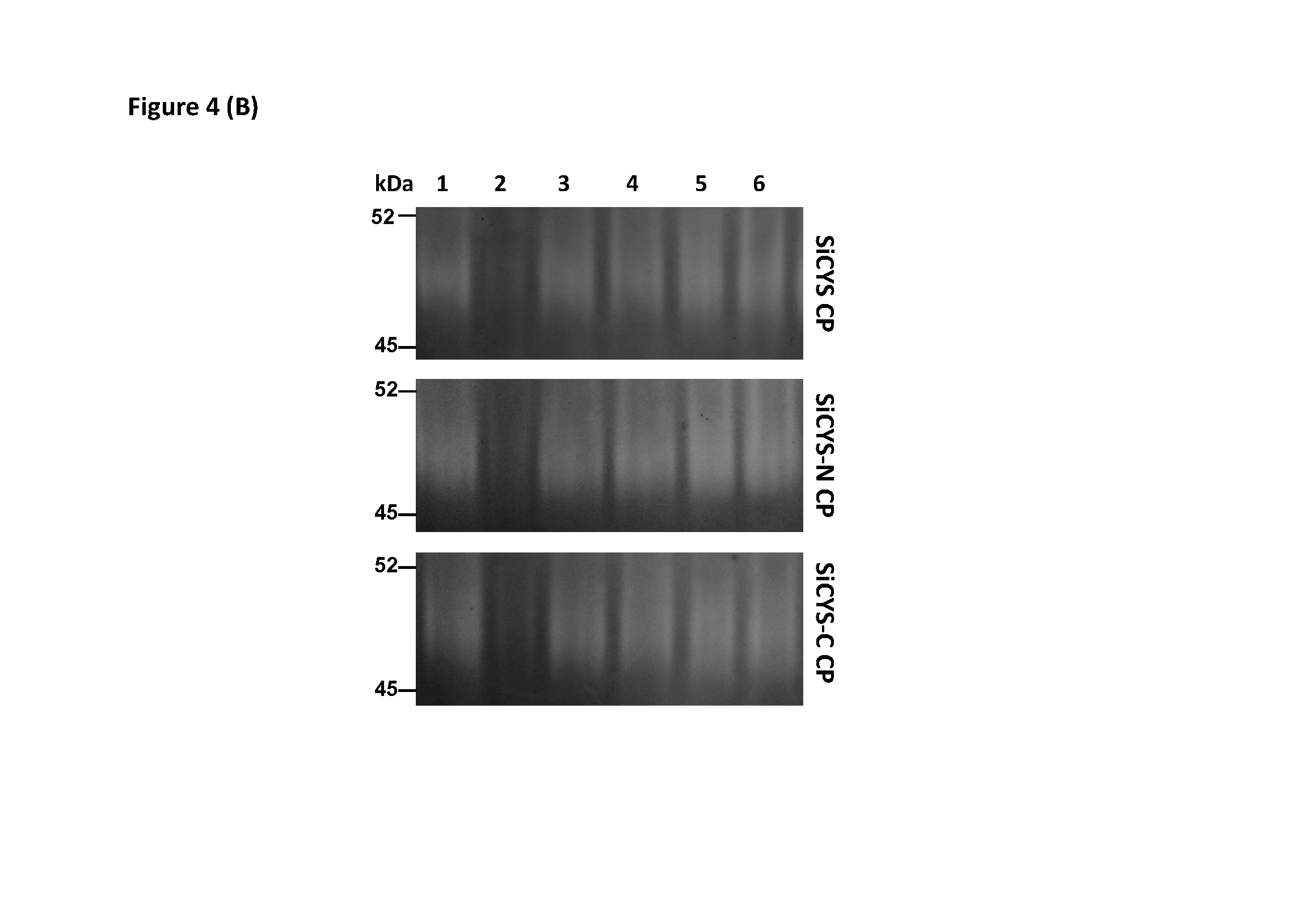

Supplement: Supplementary file 6 — Authors’ original file for figure 6 [file 40529_2013_77_MOESM6_ESM.png]

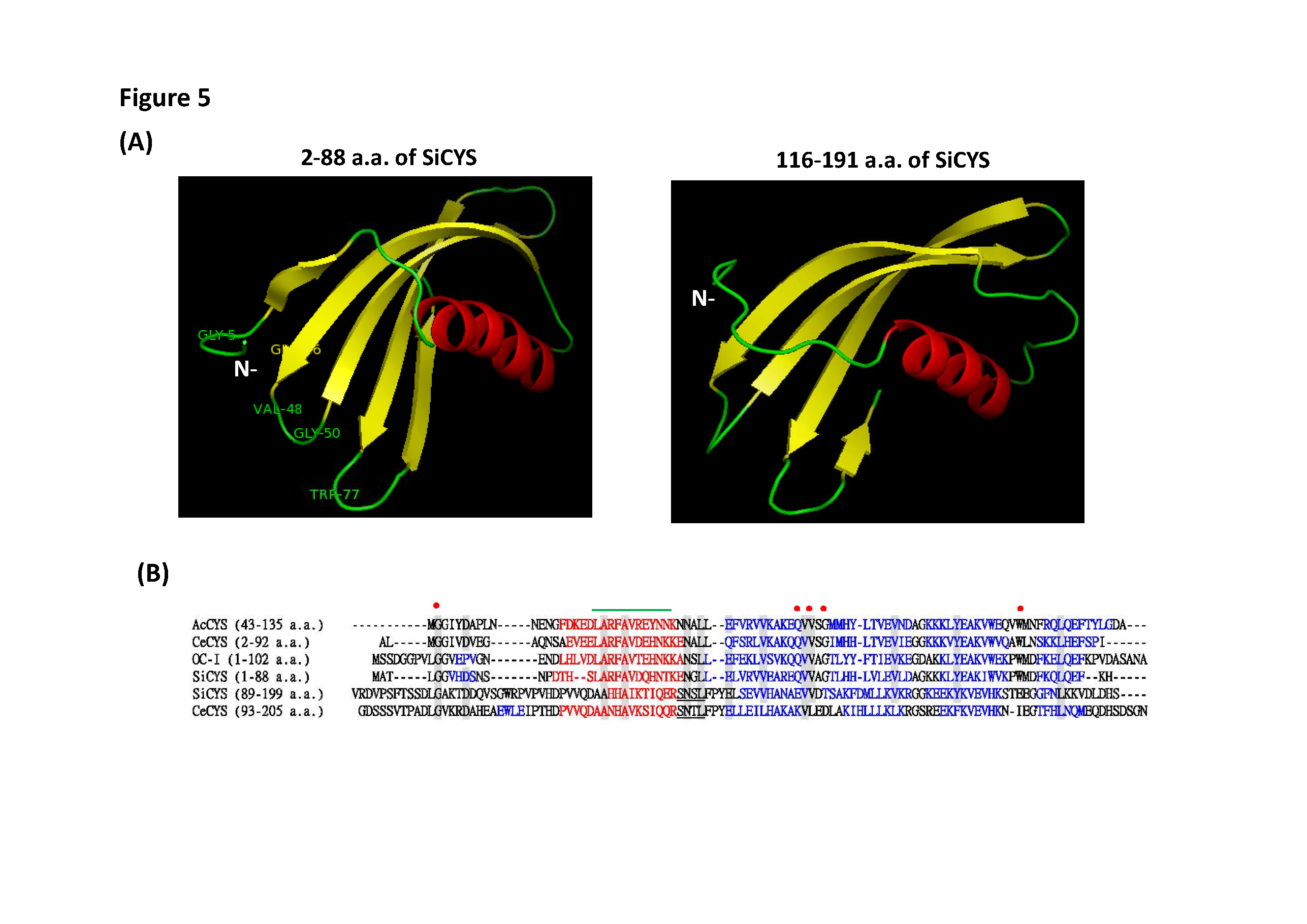

Supplement: Supplementary file 7 — Authors’ original file for figure 7 [file 40529_2013_77_MOESM7_ESM.png]
